# Team-based digital communication reduced patient-initiated phone calls to the hospital and improved patient satisfaction after orthopedic surgery: a randomized controlled trial in 70 patients

**DOI:** 10.2340/17453674.2024.40707

**Published:** 2024-05-17

**Authors:** Lili Worre Høpfner JENSEN, Søren KOLD, Birthe DINESEN, Hans-Christen HUSUM, Regitze Gyldenholm SKALS, Søren Peter EISKJÆR, Rasmus ELSØE, Ole RAHBEK

**Affiliations:** 1 Interdisciplinary Orthopaedics, Department of Orthopaedics, Aalborg University Hospital, Aalborg; 2 Laboratory for Welfare Technologies – Digital Health & Rehabilitation, ExerciseTech, Department of Health Science and Technology, Aalborg University, Aalborg East; 3 Research Data and Biostatistics, Aalborg University Hospital, Aalborg, Denmark

## Abstract

**Background and purpose:**

Post-discharge inquiries to the hospital are predominantly conducted through phone calls. The rigid timing of these calls is inconvenient for patients and disrupts the workflows of healthcare professionals. The aim of this study was to investigate the effect of a team-based digital communication intervention (eDialogue) facilitated through a messenger-like commercial solution on patient-initiated phone calls to the hospital after discharge. Secondarily, we investigated other patient-initiated contacts, patients’ perception of continuity of care, and their perception of feeling safe and satisfied after hospital discharge.

**Methods:**

On the day of discharge, 70 surgically treated orthopedic patients were randomized to the intervention group with access to eDialogue (n = 35) or the control group with standard communication pathways by phone call (n = 35) for the following 8 weeks. Through eDialogue, the intervention group had access to team-based asynchronous digital communication in text and photos with healthcare professionals across disciplines and sectors. Inclusion criteria were discharge to own home and receipt of rehabilitation services from both hospital and primary care after discharge.

**Results:**

We found a significant reduction in the mean number of patient-initiated phone calls to the hospital from 2.3 (95% confidence interval [CI] 1.4–4.1) in the control group to 0.5 (CI 0.3–1.0) in the intervention group (P = 0.004). Across groups, patients reported similar perceptions of continuity of care; however, the participants in the intervention group expressed significantly improved perceptions of, and satisfaction with, access to healthcare after discharge.

**Conclusion:**

Access to eDialogue reduced patient-initiated phone calls to the hospital, enhanced patient satisfaction with healthcare accessibility, and did not compromise patients’ perception of continuity of care after discharge compared with standard communication pathways.

Effective knowledge exchange is crucial after orthopedic surgery, and involves patients, relatives, and healthcare professionals spanning various disciplines and sectors [1]. Nevertheless, communication deficits in the transition of care from hospital to home are well-known challenges [2]. The Danish healthcare system, publicly funded and jointly managed by regions, municipalities, and general practice [3], faces a persistent issue of insufficient information exchange, contributing to adverse events that ultimately compromise patient safety [4].

Patients experience challenges in contacting healthcare professionals after hospital discharge, a situation they find inconvenient, unsafe, and frustrating [5]. Similarly, healthcare professionals experience post-discharge phone calls as time-consuming and interruptive to their workflows [6].

In recent years, digital patient platforms and secure messaging have increasingly been used between patients and hospital staff [7], but are not yet fully implemented or adopted [8]. In the context of early discharge with expectations for patients and municipal providers to handle more of the postoperative period at home [9], the availability of communication with hospital specialists has not kept pace. In contrast to other digital patient platforms facilitating patient–provider communication, we suggest that a team-based and cross-sectoral approach to digital communication involving the patient as a partner may reduce post-discharge phone calls and enhance patient satisfaction. Therefore, the aim of this study was to investigate the effects of a team-based digital communication intervention (eDialogue) facilitated through a messenger-like solution (“LetDialog,” Visma, Aarhus, Denmark) primarily on the number of patient-initiated phone calls to the hospital after discharge. Secondarily, we investigated other patient-initiated contacts, patients’ perception of continuity of care, and their perception of feeling safe and satisfied after discharge from the hospital.

## Methods

### Study design

This study was a multidisciplinary, cross-sectoral, 2-arm, open-label, randomized controlled trial. Reporting followed the CONSORT 2010 Statement: Updated guidelines for reporting parallel group randomized trials [10] and was registered at ClinicalTrials.gov [11].

### Setting and participants

Participants were included from the orthopedic surgery department at Aalborg University Hospital, Denmark, based on eligibility criteria listed in Table 1.

**Table 1:** Eligibility criteria

**Inclusion criteria**
Participants of all ages who were (or had a child who was): Hospitalized for more than 24 hours. Undergoing planned or acute trauma- or deformity-correcting orthopedic surgery. Discharged to their own home. In need of cross-sectoral care after discharge (e.g., follow-up at the outpatient clinic complemented by municipal physiotherapy or district nursing).
**Exclusion criteria**
Participants who were (or had a child who was): Previous users of eDialogue in a qualitative study [5]. Discharged to a healthcare center, nursing home, or similar. Not speaking or reading Danish or English well enough to understand participant information and to use digital communication in texts.

Recruitment took place from November 1, 2022 to March 6, 2023. For patients under 18, a parent participated on their behalf. Healthcare professionals from Aalborg University Hospital and collaborating municipalities were recruited prior to study start or ad hoc when their participation in eDialogue was wanted by participants with access to eDialogue.

### Study flow, randomization, and blinding

After inclusion participants were randomized 1:1 to standard communication pathways or eDialogue using the REDCap randomization tool [12], and in randomly concealed block sizes of 2–4. As we assumed that communication needs after discharge would differ depending on whether the admission was planned or acute, we stratified participants according to this.

Owing to the nature of the intervention, blinding was not possible. Outcome data was self-reported by participants digitally from their homes. All patients were to receive the usual standard care after hospital discharge. Data collection for each individual participant was 8 weeks after discharge, as the majority of orthopedic surgery patients have follow-up appointments in the outpatient clinic up to 6–8 weeks after discharge. This time frame encompassed the immediate vulnerable period following discharge and had also been examined in prior studies of the intervention [5,6]. If participants did not respond to the questionnaire for the primary outcome within 3 days, reminder phone calls were initiated. Participants were notified of this in advance of their inclusion.

### Intervention and procedures

The intervention was named eDialogue and involved access to team-based digital communication with healthcare professionals participating in the patient’s treatment and care and lasted 8 weeks after hospital discharge. Healthcare professionals included, for example, the orthopedic surgeon, a nurse and a secretary from the outpatient clinic, and a physiotherapist from the hospital or municipality.

eDialogue was facilitated through a messenger-like solution owned by a private company (Visma) [13]. Participants and the healthcare teams could communicate asynchronously in a shared chat and, thus, if they needed contact after discharge, communication could occur directly through text and photos in eDialogue. The research team selected the technical solution for communication prior to the study, considering its simple and intuitive interface. The solution was evaluated by patients and healthcare professionals during an initial workshop held before the study and in qualitative studies [5,6]. eDialogue was an add-on to standard communication pathways, and participants could choose to use it based on their individual preferences. After randomization to the intervention group, participants were introduced to the use of eDialogue by the recruiting research team member (Table 2 and Figure 2, see Appendix). No regular pre-training was conducted as the technical solution was found intuitive to use in prior studies [5,6]. The digital dialogues were monitored on a daily basis by a project group member and stored in a secure, cloud-based solution [13] as well as in the EHR.

**Figure 2:**
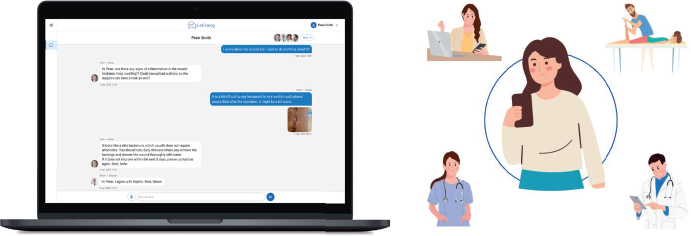
The eDialogue intervention consisted of access to team-based digital communication between patients and healthcare professionals across disciplines and sectors.

**Table 2:** Procedures for participants in the intervention group

Timeline
Day of discharge after inclusion and randomization
1. Help to download app for smartphone and create user with digital signature. Finger touch or face recognition could be used to log in. Access was also available through web.
2. Thorough introduction to the use of eDialogue for non-acute issues after discharge, including use of text, photos, and notification of new posts.
3. Participants were told to expect a 24-hour response time during weekdays. On weekends and public holidays, participants could not expect a response until the next weekday, and thus were advised to call instead.
4. In the event of an urgent need for contact, they were explicitly advised to contact by phone call.
5. Inclusion of healthcare professionals involved in the individual patient’s care pathways across sectors was identified in collaboration with participants.
Weeks 1–2
6. If the participants did not use eDialogue in the first week, a short message was sent to ensure that they could use the solution and that they had no technical challenges preventing use.
7. In the event that the rehabilitation site was unknown on the day of discharge, the physiotherapist from the municipality was included at week 1–2.
Week 8
8. 3 days before the end of the study period, the participants were informed that eDialogue would be closed.

### Standard communication pathways

Participants randomized to the control group used standard communication pathways. At Aalborg University Hospital, standard communication pathways for patients after hospital discharge adhere to the Administrative Health Agreement stating that patients who need contact within 72 hours of discharge are advised to call the hospital ward from which they were discharged. This is typically done through a main number for the hospital and involves several intermediaries. Telephone counseling can be provided by the ward nurses available at the time of calling. If serious conditions are suspected, an orthopedic surgeon or the emergency department will be contacted by the nurse for assessment, triage, and possibly readmission. Patients who need contact after 72 hours from discharge, and who are scheduled for follow-up visits, are advised to call the outpatient clinic by phone during designated phone hours.

Communication between healthcare professionals across sectors after a patient has been discharged takes place through the various electronic health records (EHRs) implemented in hospitals, municipalities, and by general practitioners. This involves exchange of text-based documents such as discharge letters, patient care plans, rehabilitation plans, and electronic correspondence [3]. However, a significant quantity still relies on traditional phone calls, and as patients are not part of the EHR communications, they communicate with healthcare professionals primarily by phone [5].

### Outcomes, data collection, and measurements

All outcomes were described at study registration at clinicaltrials.gov [11]. The primary outcome was the number of patient-initiated phone calls to the hospital 8 weeks after discharge. Secondary outcomes were other patient-initiated contacts with the hospital and other healthcare facilities through email, video, or SMS, patients’ perception of continuity of care measured by the Patient Continuity of Care Questionnaire (PCCQ) [14], and patients’ perception of feeling safe and satisfied after hospital discharge assessed by a short self-developed questionnaire (Supplementary data). Participants self-reported outcome data digitally from their homes. They received a link to the REDCap database via SMS to facilitate easy data collection.

The baseline assessments included sociodemographic and clinical data, including type of admission (planned or acute), type of surgery (deformity correction or trauma including spinal fractures), and comorbidities. Length of hospital stay and readmissions during the study period were retrieved from health records at the end of the study period.

The primary outcome was assessed through a weekly questionnaire, comprising 1 initial question asking if the patient had made any contact with healthcare professionals regarding their recent surgery during the last week. If yes, follow-up questions were asked about where to, which modality was used (phone calls, email etc.), and how many times.

Patients’ perception of continuity of care was measured by the PCCQ [14]. PCCQ is a generic questionnaire developed to cover the 3 dimensions of continuity of care defined by Haggerty et al. [15]. It is designed to assess patients’ perception of continuity of care both before and after hospital discharge [14]. On the day of discharge, participants completed the “before discharge” part of PCCQ (item 1–27). This served as a baseline to assess any group imbalances in perceptions of continuity of care at baseline. 4 weeks later, participants completed the remaining part of PCCQ concerning “after discharge” (item 28–41).

8 weeks after discharge, a 4-item questionnaire was distributed to assess patients’ perceptions of feeling safe and satisfied with access to healthcare professionals.

Ultimately, in an exploratory analysis, we asked participants in the intervention group to state their preferences regarding communication modality, to rate the extent to which they had appreciated eDialogue, and whether it had made the post-discharge period easier. Moreover, we examined usage data of eDialogue, including how many healthcare professionals were involved in eDialogue, the proportion of patients or parents who used eDialogue, and how many questions they had initiated during the trial period.

### Sample size

Prior to this study, we did not have representative numbers of patient-initiated phone calls after discharge. Therefore, we based the sample size calculation on the available knowledge, which was the proportion of patients with access to eDialogue who called the hospital after discharge in a previous qualitative study (18%) [5], and the proportion of patients who called the hospital in a random sample of patients coming to the outpatient clinic for planned follow-up 6–8 weeks after discharge (63%). Thus, drawing on this, we assumed the mean number of phone calls in the intervention and the control groups to be 0.18 and 0.63, respectively, during 8 weeks of follow-up. Based on the Poisson distribution and with a significance level at 5% and a power of 80%, the sample size was 32 in each group. To account for unexpected dropout and unequal distribution of randomization, we aimed to include a total of 70 patients for randomization.

### Statistics

All data analysis was conducted with R (v. 4.2.2) (R Foundation for Statistical Computing, Vienna, Austria) by a statistician (RS) who received blinded data for the primary and secondary outcomes. All tests were 2-sided, and the significance level was set to 5%. Categorical variables were presented with count and percentages, and compared between intervention and control group using Fisher’s exact test. After examination for normality using QQ plots, age was presented with mean and standard deviation, and length of hospital stay was presented by median and interquartile range (IQR).

The mean number of phone calls to the hospital was compared between the intervention and the control group, using a negative binomial regression model with the link function being “identity”. The link function “identity” was chosen to calculate the absolute difference in the mean number of phone calls between the intervention and the control group. In the model, only a variable indicating the study group (intervention/control) was included. This model was chosen due to a large number of patients who did not call the hospital after discharge, resulting in many zero counts and an over-dispersed Poisson regression model. Over-dispersion in the Poisson regression model was detected by computing the residual deviance divided by the degrees of freedom of the model.

PCCQ and satisfaction data was non-normally distributed and presented by means and bootstrapped 95% confidence intervals (CI). Data was compared by estimating the mean difference between the intervention and the control group. The percentile method was used to estimate confidence intervals from the bootstrapped data based on 1,000 iterations. Data in the response category N/A was treated as missing data.

### Ethics, registration, data sharing plan, funding, and disclosures

Written informed consent was collected from all participants prior to inclusion and the study complied with the Declaration of Helsinki. As per response by the Scientific Ethics Committee of North Denmark Region, the study did not require ethical approval (journal number: 2021-000438). The study was registered at Aalborg University Hospital registry for research (ID number: 2021-219) and at clinicaltrials.gov [11]. A data processor agreement was made between the provider of eDialogue (Visma) and Aalborg University Hospital. Helsefonden and the North Denmark Region supported the study. Data sharing may be permitted by request to the first author. The authors declare no conflicts of interest. Complete disclosure of interest forms according to ICMJE are available on the article page, doi: 10.2340/17453674.2024.40707

## Results

### Participant characteristics

Of 165 patients assessed for eligibility, 73 were eligible for inclusion, and all but 3 consented to participate. 35 were randomized to the control group and 35 were randomized to the intervention group (Figure 2).

**Figure 2:**
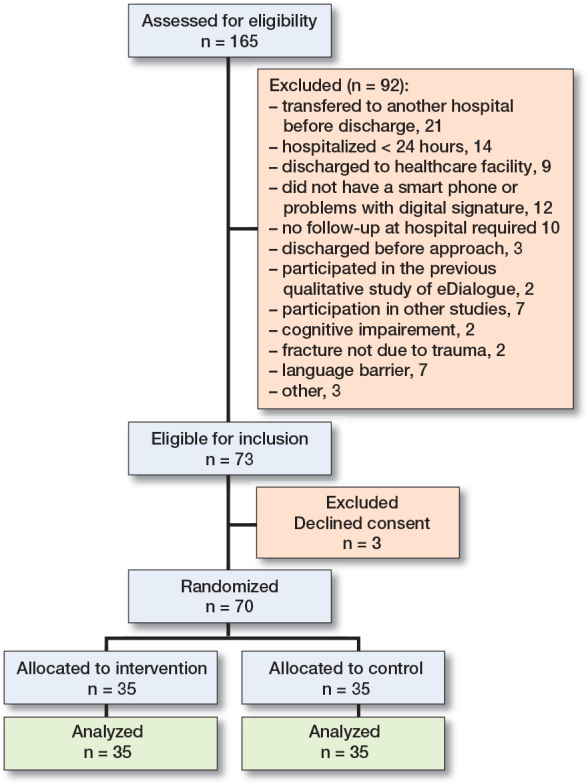
Participant flow diagram and reasons for exclusion.

There were no dropouts; however, 9 patients were contacted between 1 to 3 times to remind them to answer the questionnaires. This resulted in a 100% response rate.

The 3 patients who declined consent after being screened and found eligible were 2 male and 1 female aged 15, 23, and 28 years. Of these, 2 of them were admitted for planned surgery and 1 was admitted acutely. The reason for refusal was a lack of capacity to answer questionnaires during the study period.

All patients who were under 18 years of age wanted a parent to participate on their behalf. Across the groups this was equally distributed (37% vs. 26%) (Table 3).

**Table 3:** Baseline sociodemographics of participants (patients or parents, if the patient was < 18 years old) presented by groups. Values are count (%) unless otherwise specified

Factor	Control Group (n = 35)	Intervention group (n = 35)
Participant on behalf of their child (parent)	13 (37)	9 (26)
Admission type		
Planned surgery	15 (43)	15 (43)
Acute admission	20 (57)	20 (57)
Length of hospital stay, median (IQR)	5 (3.0–6.5)	5 (3.0–7.0)
Readmissions during follow-up	2 (6)	3 (9)
Total length in days, mean (range)	26 (7–45)	17 (3–36)
Sex		
Female	20 (57)	25 (71)
Male	15 (43)	10 (29)
Age, mean (SD)	46 (14)	44 (15)
Cohabitation status		
Living alone	6 (17)	6 (17)
Cohabiting	29 (83)	29 (83)
Highest completed level of education		
Primary and high school	9 (26)	6 (17)
Vocational education	9 (26)	7 (25)
Higher education ^a^	17 (49)	22 (63)
Current occupation		
Student or working	28 (80)	26 (74)
Available to work or retired	7 (20)	9 (26)
Daily use of smartphone or the internet		
via computer or tablet, n (%)	35 (100)	35 (100)

a Short, bachelor’s and master’s degree.

The 2 groups were similar in age (46, SD 14 vs. 44, SD 15) and sex distribution (female 57%, male 43% vs. female 71%, male 29%). Likewise, the groups were equally distributed in relation to admission type (planned 43% and acute 57% in both groups) and length of hospital stay (median 5, IQR 3.0–6.5 vs. median 5, IQR 3.0–7.0). There were 2 readmissions during follow-up in the control group and 3 in the intervention group of varying length (Table 3).

### Primary outcome

#### Patient-initiated phone calls to the hospital after discharge

8 weeks after discharge, participants in the control group had reported 82 phone calls to the hospital versus 17 in the intervention group corresponding to 2.3 (CI 1.4–4.1) in the control group and 0.5 (CI 0.3–1.0) in the intervention group (Table 4). The difference between the mean number of phone calls in the control group and in the intervention group was a significant reduction of –1.9 (CI –3.6 to –0.9). Moreover, there was a smaller proportion of participants who had called after discharge in the intervention group by 31% compared with 60% in the control group (P = 0.03) (Table 4).

**Table 4:** Number and proportion of patient-initiated phone calls to the hospital during 8 weeks after discharge

Place at hospital	Number of calls n		Proportion of participants who called at least once n (%)	
	Control group (n = 35)	Intervention group (n = 35)	Control group (n = 35)	Intervention group (n = 35)
Outpatient clinic	40	7	11 (31)	6 (17)
Hospital ward	26	3	13 (37)	2 (5.7)
Physiotherapy department	12	3	6 (17)	2 (5.7)
Out-of-hours doctor	2	2	2 (5.7)	2 (5.7)
Emergency department	1	1	1 (2.9)	1 (2.9)
Directly to surgeon ^a^	1	1	1 (2.9)	1 (2.9)
Total n,	82	17	21 (60) ^c^	11 (31) ^c^
mean	2.3 ^b^	0.5 ^b^		
CI	1.4–4.1	0.3–1.0		

a Directly to the surgeon indicates cases where the participant was given the surgeon’s direct phone number in case contact was needed post-discharge. This is not a standardized procedure.

b P = 0.004.

c P = 0.03.

The distribution of phone calls per group and the mean number of phone calls over the 8 weeks can be accessed in Supplementary data.

### Secondary outcomes

#### Contacts other than phone call and contacts with other healthcare facilities

3 participants from the control group and 1 participant in the intervention group had contacted the surgeon. Video consultations were reported by 1 participant from the control group and 3 from the intervention group.

Participants from both groups also contacted the general practitioner, home care, and physiotherapy in the municipalities (Table 5).

**Table 5:** Proportion of participants who contacted other healthcare facilities outside the hospital 8 weeks after discharge. Values are count (%)

Contacts with other healthcare facilities	Control group (n = 35)	Intervention group (n = 35)
General practitioner	7 (20)	3 (8.6)
Home care or home care nursing, municipality	1 (2.9)	2 (5.7)
Physiotherapy, municipality	3 (8.6)	4 (11)
Total	9 (26)	7 (20)

No significant difference was found between the groups’ contacts with healthcare professionals outside the hospital.

#### Patients’ perception of continuity of care

There was no significant difference in PCCQ between groups at baseline, indicating that the groups had similar perceptions. 4 weeks after discharge, we found no significant difference between the groups’ PCCQ (Table 6). The full report of the PCCQ questionnaire can be assessed in Supplementary data.

**Table 6:** Perception of continuity of care at baseline and 4 weeks after discharge measured by PCCQ. Values are mean (CI)

PCCQ items	Control group (n = 35)	Intervention group (n = 35)	Difference
at discharge,	4.6	4.4	–0.2
items 1–27	(4.5–4.7)	(4.2–4.6)	(–0.4 to 0.1)
4 weeks after discharge,	4.1	4.2	0.1
items 28–41	(3.9–4.4)	(4.0–4.4)	(–0.2 to 0.4)

#### Patients’ perception of feeling safe and satisfied after discharge

8 weeks after discharge, participants in the intervention group reported a significantly increased perception of knowing whom to contact regarding post-discharge questions. Moreover, they experienced that it was easier to get in contact with healthcare professionals and they reported being significantly more satisfied with their opportunities for contact after discharge compared with the control group (Table 7).

**Table 7:** Participants’ perception of feeling safe and satisfied 8 weeks after discharge. Values are mean (CI)

Questions	Control group (n = 35)	Intervention group (n = 35)	Difference
1. I feel secure after discharge	4.5 (4.3–4.7)	4.6 (4.3–4.8)	0.1 (–0.2 to 0.4)
2. I know whom to contact if I have questions after discharge	4.3 (4.1–4.6)	4.7 (4.6–4.9)	0.4 (0.1 to 0.7)
3. It is easy to get in touch with the healthcare professionals who know me (e.g., surgeon, nurse, physiotherapist)	4.0 (3.7–4.3)	4.7 (4.6–4.9)	0.7 (0.4 to 1.0)
4. I am satisfied with my oppor tunities to get in touch with health-care professionals after discharge	4.1 (3.8–4.4)	4.7 (4.6–4.9)	0.6 (0.3 to 1.0)

### Exploratory analysis

#### Use of eDialogue

6 healthcare professionals, including 10 orthopedic surgeons, 3 secretaries and 4 nurses from the outpatient clinic, and 9 physiotherapists from the hospital or municipalities, were connected with participants in eDialogue. Overall, 26 of 35 participants (74%) had used eDialogue 8 weeks after discharge. These participants initiated 114 dialogues (mean 5.6; range 1–11), of which 103 (90%) were formulated as a question needing a reply from healthcare professionals, primarily aimed at and answered by hospital staff.

#### Evaluation of eDialogue by the intervention group

When participants in the intervention group were asked about their preferred communication modality, 91% rated eDialogue as their favorite over phone call, email, or video consultation. The general evaluation by the intervention group was positive (Table 8).

**Table 8:** Evaluation of access to eDialogue rated by participants from the intervention group. Values are count (%)

Questions	Likert-like scale ^a^				
	1	2	3	4	5
1. I appreciated the opportunity to use eDialogue for communication with healthcare professionals after hospital discharge	1 (2.9)	0 (0)	1 (2.9)	4 (11)	29 (83)
2. To be able to contact healthcare professional through eDialogue has made my care pathway easy	1 (2.9)	0 (0)	3 (8.6)	8 (23)	23 (66)

a Participants stated their level of agreement in 5 points: 1 = strongly disagree; 2 = disagree; 3 = neither agree nor disagree; 4 = agree; and 5 = strongly agree.

All the participants who responded 1–3 on the scale for question 1 were among those who did not use eDialogue at all during the study period. 4 of the participants who responded 1–3 for question 2 did not use eDialogue.

## Discussion

This study demonstrated that access to eDialogue significantly reduced the volume of patient-initiated phone calls to the hospital 8 weeks after discharge compared with standard communication pathways by approximately 2 calls per patient. Thus, post-discharge phone calls could be moved to asynchronous digital communication with the positive advantage of reducing the number of real-time interruptions to hospital staff. This is of clinical importance, because working life in orthopedic departments is already time-constrained and, as healthcare professionals perform complex tasks, interventions aiming to minimize interruptions are imperative. While participants in the intervention group made fewer phone calls following discharge, some calls persisted. This indicates that completely eliminating phone calls may not be feasible, and that both communication modalities may be beneficial as complementary options. However, as patients were instructed to call if they deemed their inquiry to be urgent, we did not anticipate that access to eDialogue would entirely replace phone calls.

In previous literature, a decrease in the number of phone calls when patients are provided with access to an online messaging platform has been suggested [16]. However, others have reported the opposite or no difference [17,18]. Due to substantial heterogeneity of studies within this domain, systematic reviews have encountered challenges in reaching definitive conclusions regarding effects on resource consumption, including phone calls [19,20]. In clinical practice, phone inquiries to hospitals are not registered systematically, and call management varies across settings, which also poses a challenge for comparable studies [7]. Therefore, this study adds to the sparse knowledge concerning the effect of asynchronous digital communication between patients and healthcare professionals in the period after discharge.

We showed a significant difference in proportions of participants who had initiated phone calls to the hospital after discharge. However, a full 74% of the intervention group had used eDialogue, while 60% of participants in the control group had initiated contact. This may indicate an increase in patients making contact when given the opportunity to do so through a digital solution, which should be taken into account prior to implementing such solutions.

An advantage of eDialogue is that healthcare professionals with the optimal domain knowledge can provide a high-quality response directly to the patient. This is in contrast to phone calls, where there are several intermediaries. However, text messages may take place over a period of time when engaging in multiple back-and-forth exchanges and thus be disruptive in another way. Since we did not measure the time needed for healthcare professionals to answer messages versus time used on answering phone calls, we cannot conclude that eDialogue reduces the workload of hospital staff, even though it may change the workflow for the better. However, there is a rationale to suspect that responding to text messages may be less time-consuming than handling phone calls. As pointed out in a systematic review of secure messaging between patients and providers, the relationship between digital patient–provider messaging and phone calls is multifaceted [21]. For example, it may be appropriate for a message to lead to a phone call, or for a phone call to be followed up via messages.

In early 2023, time needed to treat (TNT) was introduced as a novel methodological concept that puts emphasis on including healthcare staff’s time consumption in the assessment of effects [22]. The authors suggested that this outcome measure should be included in the design of, for example, clinical guidelines to make best use of sparse clinical resources. The rationale underlying this is that time spent on new procedures takes time away from other important tasks [22]. Applying this outcome measure to future studies may provide knowledge regarding the workload of healthcare professionals when introducing eDialogue.

We did not find any statistically significant difference in patients’ perception of continuity of care measured by PCCQ, indicating that this was not compromised in participants with access to eDialogue. However, several questions were answered N/A, implying a potential mismatch between the generic questionnaire and the characteristics of the patient population.

Our study demonstrated a significant increase in patients’ perception of ease of access and satisfaction with access after discharge. Increase in patient satisfaction has been reported in other studies where patients were given access to text-based digital communication, including a systematic review on the impact of patient portals [23]. In orthopedic surgery, perioperative anxiety is a major concern affecting postoperative outcomes [24], and increased access to healthcare may enhance the support of patients, an important factor in the context of early discharge.

Consistent with the trend towards patients accepting digitized healthcare services, participants in the intervention group reported preferences to use eDialogue for post-discharge communication. However, based on the quantity of patient-initiated inquiries in both groups, our study emphasizes a need for change that may also involve efforts aimed at enhancing patient education and data sharing between patients and healthcare professionals across sectors. Initiatives to improve patient education and data sharing can, for example, be the development of patient-facing digital platforms [23], enhanced sharing of discharge letters and outpatient visit notes [25,26] and personally collected health data [27].

### Limitations

Due to the intervention of this study, we could not blind either patients, parents, or healthcare professionals. Since the majority of participants had a clear preference for being randomized to the intervention group, this may have influenced their answers regarding safety and satisfaction. Moreover, participants in the intervention group were contacted in the first week after discharge if they had not used eDialogue and may thus have been subjected to attention bias. It is likely that it was in favor of the intervention group and that it has resulted in performance bias and a systematic difference between the groups. Also, the questions were formulated with a positive tone, and as patient satisfaction surveys are sensitive to the wording of questions, this may have affected the strength of agreement with statements. However, a recent study of patient satisfaction surveys in the outpatient clinic demonstrated that disagreement with a negatively toned question was stronger than agreement with a positively toned question [28].

At the end of the trial, participants in the intervention group were asked about their preferences regarding communication modality, rate of appreciation, and whether they felt access to eDialogue had made the post-discharge period easier. Although this analysis is exploratory, it can be criticized as there is no basis for comparison.

### Conclusion

Access to eDialogue reduced patient-initiated phone calls to the hospital during 8 weeks by 2 calls per patient after discharge and increased patient perceptions of ease of access and satisfaction with accessibility to healthcare after hospital discharge.

In perspective, there seems to be untapped potential in using team-based digital communication in orthopedic surgery care pathways; however, future studies should explore additional measures of effect and economic incentives.

### Supplementary data

The self-developed questionnaire, distribution of phone calls, and full PCCQ data are available as Supplementary data on the article page, doi: 10.2340/17453674.2024.40707

## Supplementary Material



## Notes

LJ, OR, SK, BD, RE, RS, SE, and H-CH contributed to the study design; LJ collected data in collaboration with a research nurse; LJ and RS planned the statistical analysis, later performed by RS; all authors contributed to interpreting the results and revising the manuscript.

Thanks are offered to all participants and healthcare professionals involved in the study across sectors, and to the dedicated study nurse, Tina Lyngholm Jensen, for coordinating and assisting in the recruitment of patients.

Handling co-editors: Li Felländer-Tsai and Philippe Wagner

*Acta* thanks Bergita Ganse and Leif Rune Hedman for help with peer review of this manuscript.
