# Supplementary material for: Team-based digital communication reduced patient-initiated phone calls to the hospital and improved patient satisfaction after orthopedic surgery: a randomized controlled trial in 70 patients
Source: Acta Orthop. 2024 May 17;95:225–32. doi: 10.2340/17453674.2024.40707 (PMC11100491; doi:10.2340/17453674.2024.40707)
Supplement: Supplementary file 1 [file ActaO-95-40707-s001.pdf]

Supplementary material

Assessment of outcomes

All questionnaires used in this study were self-reported by patients. Questionnaires distributed before discharge were collected on a tablet at the hospital ward, and questionnaires after discharge were distributed by SMS with a link to REDCap on the participants own smartphone.

Primary outcome

The primary outcome was the number of patient-initiated phone calls to the hospital during 8 weeks after discharge. As we could not find any validated questionnaires to measure this, we developed a questionnaire prior to the study. The questionnaire was tested for wording and understanding on 12 orthopedic surgery patients coming to the outpatient clinic for follow-up 6 weeks after discharge using qualitative interviewing [1]. The questionnaire was not validated with regards to the psychometric properties.

Initially, the questionnaire included 1 question, asking *if the participant had initiated any contact to healthcare professionals within the last week* (Screenshot 1). The same questionnaire was sent to participants 1 time a week for 8 weeks after hospital discharge.

Screenshot 1.

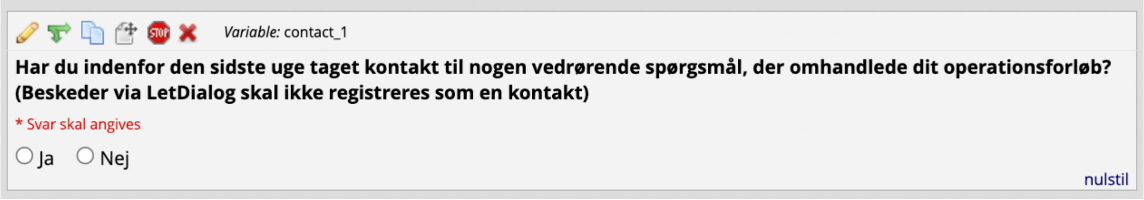
Variable: contact_1

Har du indenfor den sidste uge taget kontakt til nogen vedrørende spørgsmål, der omhandlede dit operationsforløb? (Beskeder via LetDialog skal ikke registreres som en kontakt)

* Svar skal angives

○ Ja ○ Nej

nulstil

If participants answered “Nej” (Danish for “no”), no other questions would be asked.
If participants answered “Ja” (Danish for “yes”), another question would occur, asking *whom have you contacted/where to* (Screenshot 2).

Screenshot 2.

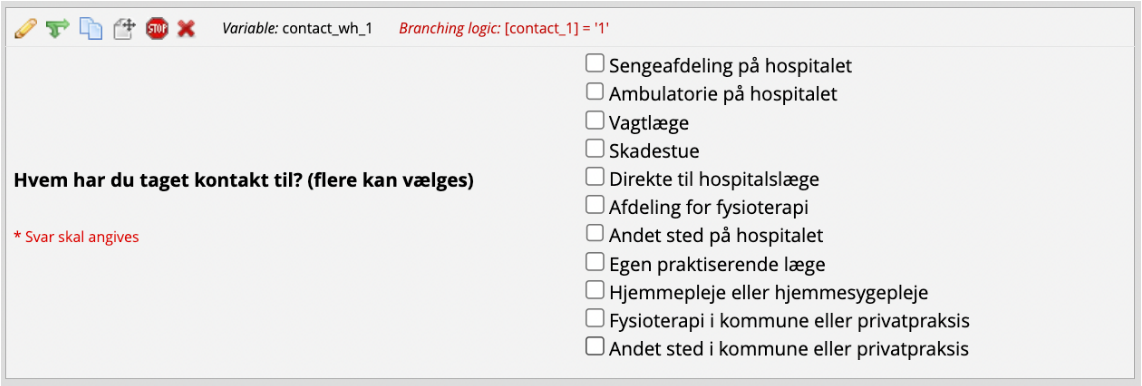
Variable: contact_wh_1 *Branching logic:* [contact_1] = '1'

Hvem har du taget kontakt til? (flere kan vælges)

* Svar skal angives

- ☐ Sengeafdeling på hospitalet
- ☐ Ambulatorie på hospitalet
- ☐ Vagtlæge
- ☐ Skadestue
- ☐ Direkte til hospitalslæge
- ☐ Afdeling for fysioterapi
- ☐ Andet sted på hospitalet
- ☐ Egen praktiserende læge
- ☐ Hjemmepleje eller hjemmesygepleje
- ☐ Fysioterapi i kommune eller privatpraksis
- ☐ Andet sted i kommune eller privatpraksis

Then, if participants answered any place at the hospital, for example “Sengeafdeling på hospitalet” (Danish for “ward at hospital”), they would be asked *how the contact was made* (i.e., what modality), and lastly *how many times*, they had called. The number of times was registered by choosing a number in a drop-down menu (Screenshot 3).

Screenshot 3.

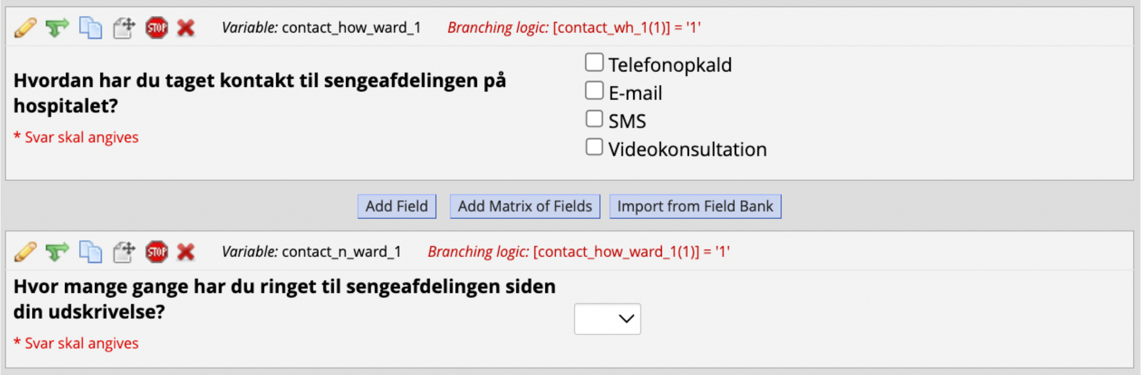

Secondary outcomes

Secondary outcomes included other contacts made by participants after discharge, patients' perception of continuity of care, and patients' perception of feeling safe and satisfied with access to healthcare after discharge. In an exploratory analysis, we assessed the use of eDialogue by the intervention group as well as participants' future preferences regarding communication modalities and ratings of access to eDialogue.

Contacts to the hospital other than by phone call and contacts to other healthcare facilities

From the questionnaire used for the primary outcome (Screenshot 1–3), knowledge on participants' contacts to the hospital made by other modalities was be collected, such as email, SMS and video consultation. Moreover, contacts made to other healthcare facilities outside the hospital, such as general practitioner, district nursing, municipal physiotherapy or other places, would be collected. This was reported as a secondary outcome, to fully grasp patient-initiated contacts throughout the healthcare system.

Patients' perception of continuity of care

The Patient Continuity of Care Questionnaire (PCCQ), developed by Hadjistavropoulos et al. (2008) [2], and based on the dimensions of Continuity of Care (COC) defined by Haggerty et al. [3], was used to assess patient's perception of continuity of care in this study. The concept of COC is used as a measure for quality of care in transitions. It encompasses *informational continuity*, which is the use of medical or personal information to provide appropriate care over time, *management continuity*, which refers to the provision of timely, coordinated and complementary services that are responsive to patients needs to connect care over time, and *relational continuity* which involves the consistency and quality of relationships between patients and providers as a means to connect care over time. All three dimensions should be achieved to ensure COC.

The original version of PCCQ was developed and psychometrically evaluated in Canada [2]. It was based upon the Heart Continuity of Care Questionnaire, adapted and tested in a geriatric and orthopedic surgery patient population. It has since then been translated and validated in other countries for different patient populations, such as patients with heart disease [4].
Answers to each question is given on a 5-point Likert scale ranging from "strongly disagree" (1 point) to "strongly agree" (5 points). Patients can also report 'not applicable' (N/A) if they do not find the questions relevant to their situation. The total score is calculated, and higher scores represent a higher degree of perception of continuity of care. The questionnaire is divided in two sections, where one involves questions regarding "before discharge" and the latter includes questions regarding "after discharge". These sections can be summarized separately. Moreover, the PCCQ can be divided into 6 subcategories, each targeting specific dimensions of COC before and after discharge (management, informational and relational COC).

Prior to this study, we translated and culturally adapted the PCCQ into Danish inspired by the principles of good practice described by Wild et al. (ISPOR) [5] and in collaboration with the developer of the questionnaire. Additional information on the translation process can be obtained upon request to the first author. A manuscript is currently being prepared for publication. An example of the translated version of the questionnaire is shown in Screenshot 4.
An integral part of the translations process involved cognitive debriefing with 10 orthopedic surgery patients comparable to patients included in this study. However, we did not test the psychometric properties of the translated version. Also, for this study the PCCQ was applied differently than originally intended. The section “before discharge” was distributed to participants on the day of discharge to provide a baseline measure that could detect differences in patients’ perception prior to randomization (Screenshot 4 shows an example of one question from the “before discharge” section). We were careful about collecting data for the “before discharge” section at the day of discharge after participants had received discharge information from hospital staff and right before leaving the hospital. The section “after discharge” was then collected four weeks after randomization and discharge (Screenshot 5 displays an example of one question from the “after discharge” section).

Screenshot 4.

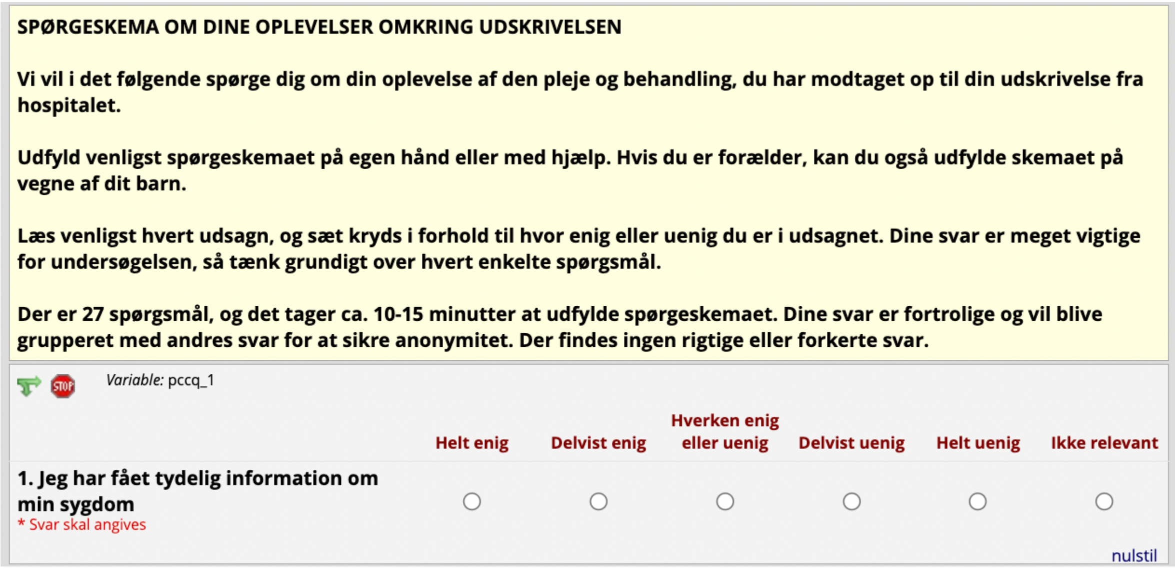
SPØRGESKEMA OM DINE OPLEVELSER OMKRING UDSKRIVELSEN

Vi vil i det følgende spørge dig om din oplevelse af den pleje og behandling, du har modtaget op til din udskrivelse fra hospitalet.

Udfyld venligst spørgeskemaet på egen hånd eller med hjælp. Hvis du er forælder, kan du også udfylde skemaet på vegne af dit barn.

Læs venligst hvert udsagn, og sæt kryds i forhold til hvor enig eller uenig du er i udsagnet. Dine svar er meget vigtige for undersøgelsen, så tænk grundigt over hvert enkelte spørgsmål.

Der er 27 spørgsmål, og det tager ca. 10-15 minutter at udfylde spørgeskemaet. Dine svar er fortrolige og vil blive grupperet med andres svar for at sikre anonymitet. Der findes ingen rigtige eller forkerte svar.

Variable: pccq_1

	Helt enig	Delvist enig	Hverken enig eller uenig	Delvist uenig	Helt uenig	Ikke relevant
**1. Jeg har fået tydelig information om min sygdom** * Svar skal angives	○	○	○	○	○	○

nulstil

Screenshot 5.

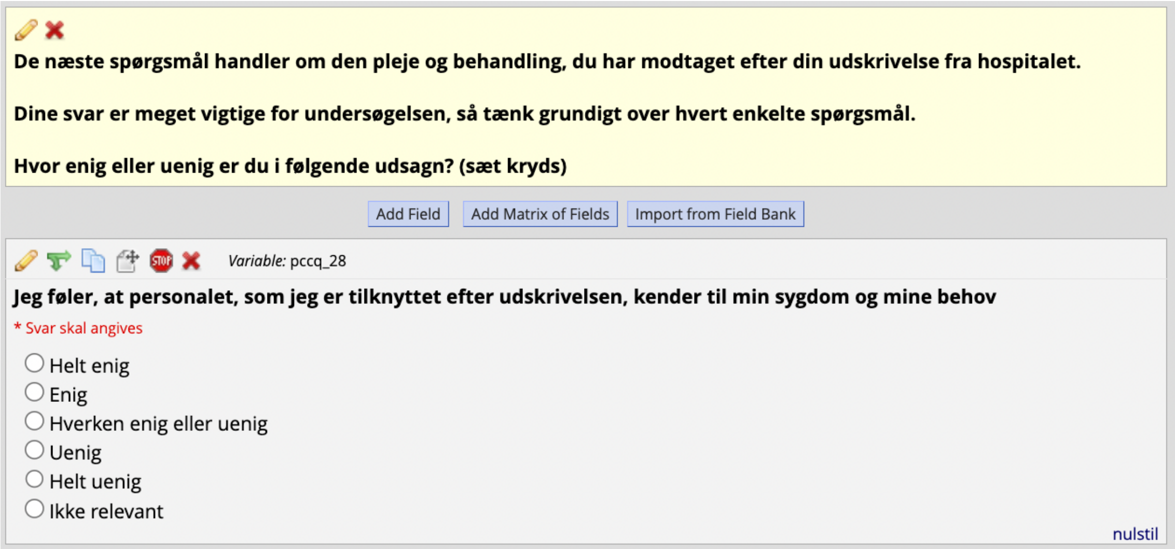
De næste spørgsmål handler om den pleje og behandling, du har modtaget efter din udskrivelse fra hospitalet.

Dine svar er meget vigtige for undersøgelsen, så tænk grundigt over hvert enkelte spørgsmål.

Hvor enig eller uenig er du i følgende udsagn? (sæt kryds)

Add Field | Add Matrix of Fields | Import from Field Bank

Variable: pccq_28

Jeg føler, at personalet, som jeg er tilknyttet efter udskrivelsen, kender til min sygdom og mine behov

* Svar skal angives

- ○ Helt enig
- ○ Enig
- ○ Hverken enig eller uenig
- ○ Uenig
- ○ Helt uenig
- ○ Ikke relevant

nulstil

Patients' perception of feeling safe and satisfied after discharge

A simple 4-item questionnaire was developed by the research team prior to the study to address patients' perception of feeling safe and satisfied with access to healthcare after hospital discharge. The questions concerned the extent to which 1) patients had been feeling safe after discharge, 2) had knowledge of whom to contact if needed, 3) experienced ease of access to healthcare, and 4) were satisfied with opportunities for contact after discharge. The questionnaire was tested for wording and understanding in 12 orthopedic surgery patients by qualitative interviewing [1]. Screenshot 6 shows the 4-item questionnaire in Danish.

Screenshot 6.

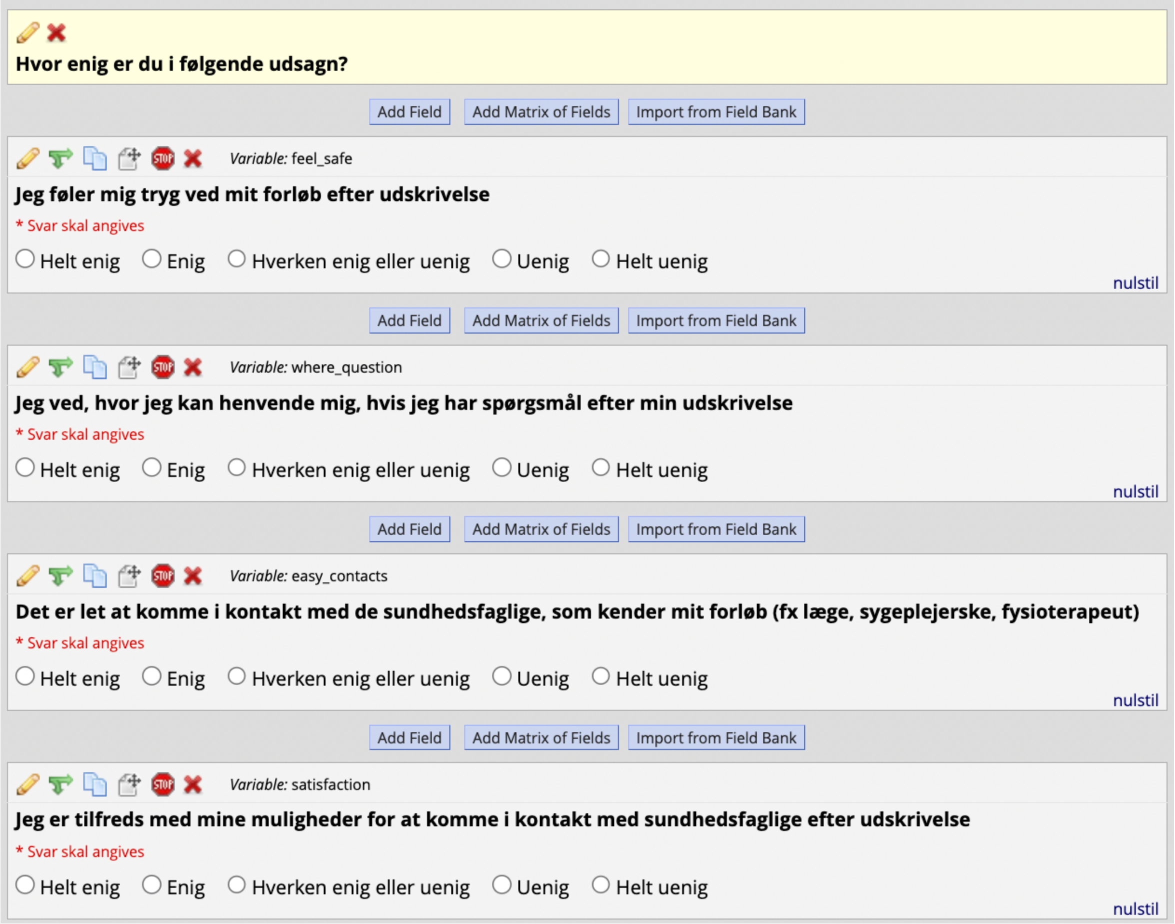

Hvor enig er du i følgende udsagn?

Add Field | Add Matrix of Fields | Import from Field Bank

Variable: feel_safe

Jeg føler mig tryg ved mit forløb efter udskrivelse

* Svar skal angives

○ Helt enig ○ Enig ○ Hverken enig eller uenig ○ Uenig ○ Helt uenig

nulstil

Add Field | Add Matrix of Fields | Import from Field Bank

Variable: where_question

Jeg ved, hvor jeg kan henvende mig, hvis jeg har spørgsmål efter min udskrivelse

* Svar skal angives

○ Helt enig ○ Enig ○ Hverken enig eller uenig ○ Uenig ○ Helt uenig

nulstil

Add Field | Add Matrix of Fields | Import from Field Bank

Variable: easy_contacts

Det er let at komme i kontakt med de sundhedsfaglige, som kender mit forløb (fx læge, sygeplejerske, fysioterapeut)

* Svar skal angives

○ Helt enig ○ Enig ○ Hverken enig eller uenig ○ Uenig ○ Helt uenig

nulstil

Add Field | Add Matrix of Fields | Import from Field Bank

Variable: satisfaction

Jeg er tilfreds med mine muligheder for at komme i kontakt med sundhedsfaglige efter udskrivelse

* Svar skal angives

○ Helt enig ○ Enig ○ Hverken enig eller uenig ○ Uenig ○ Helt uenig

nulstil

Patients' preferences for post discharge communication (only intervention group)

Eight weeks after discharge, participants in the intervention group were also asked to state what kind of contact modality they would prefer for post-discharge inquiries, choosing between eDialogue, phone call, email or video consultation (Screenshot 7), and 2), and to rate to which extent they felt eDialogue had supported them after discharge and to what extent their access to eDialogue had made the post-discharge period easier for them (Screenshot 8).

Screenshot 7. Preferences regarding contact modality by intervention group

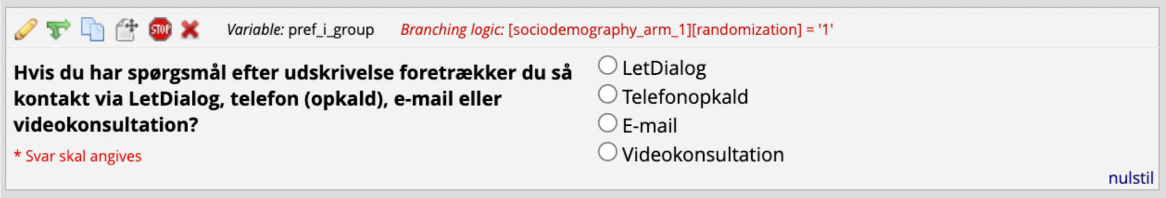
Variable: pref_i_group *Branching logic:* [sociodemography_arm_1][randomization] = '1'

Hvis du har spørgsmål efter udskrivelse foretrækker du så kontakt via LetDialog, telefon (opkald), e-mail eller videokonsultation?

* Svar skal angives

- ○ LetDialog
- ○ Telefonopkald
- ○ E-mail
- ○ Videokonsultation

nulstil

Screenshot 8. Rating of eDialogue by intervention group

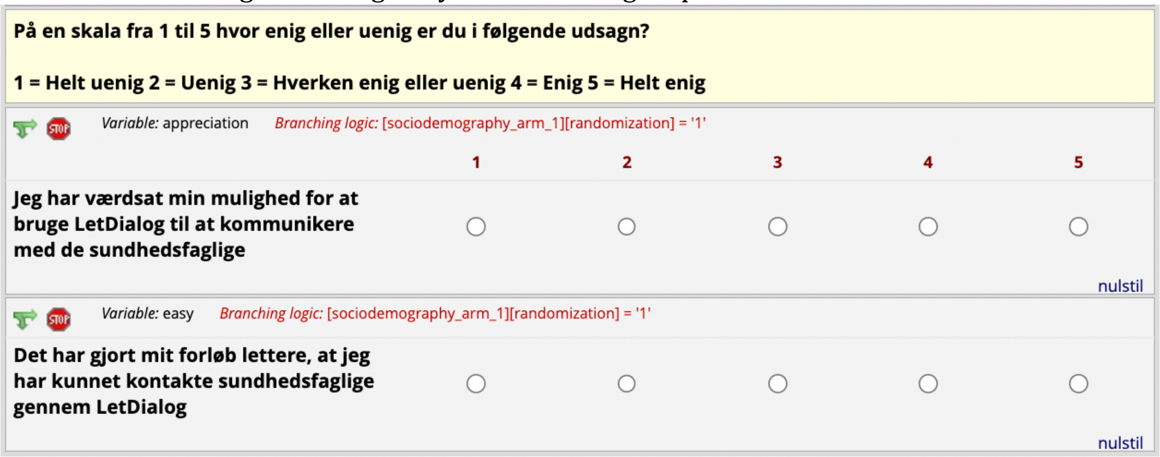
På en skala fra 1 til 5 hvor enig eller uenig er du i følgende udsagn?

1 = Helt uenig 2 = Uenig 3 = Hverken enig eller uenig 4 = Enig 5 = Helt enig

Variable: appreciation *Branching logic:* [sociodemography_arm_1][randomization] = '1'

	1	2	3	4	5
**Jeg har værdsat min mulighed for at bruge LetDialog til at kommunikere med de sundhedsfaglige**	○	○	○	○	○

nulstil

Variable: easy *Branching logic:* [sociodemography_arm_1][randomization] = '1'

**Det har gjort mit forløb lettere, at jeg har kunnet kontakte sundhedsfaglige gennem LetDialog**	○	○	○	○	○

nulstil

Primary outcome data

The complete distribution of phone calls per group presented by a density plot (Figure 1).

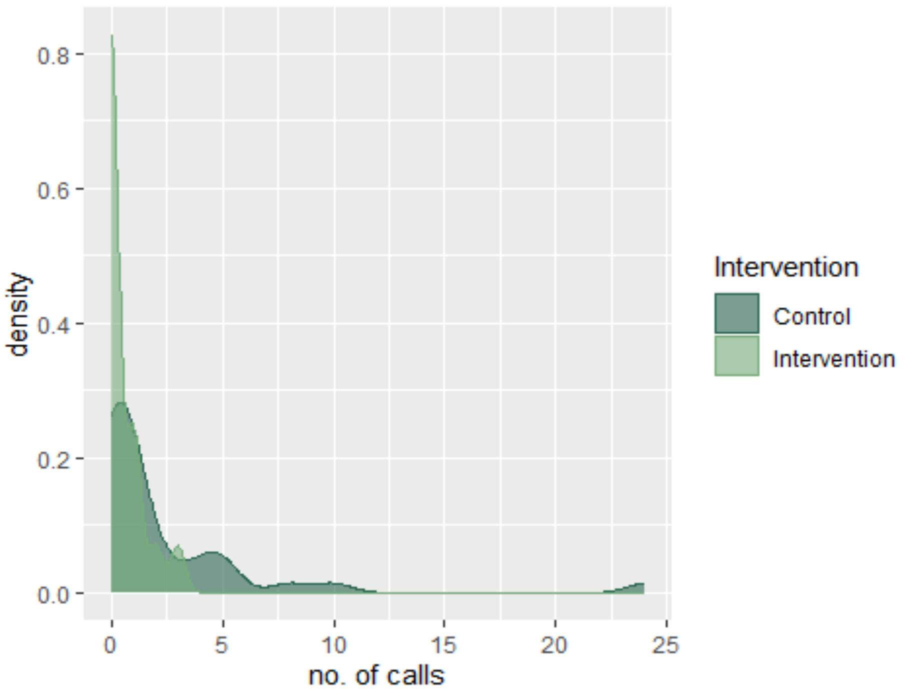

The mean number of phone calls per week by groups shows that the majority of phone calls were made within the first 2 weeks after hospital discharge (Figure 2).

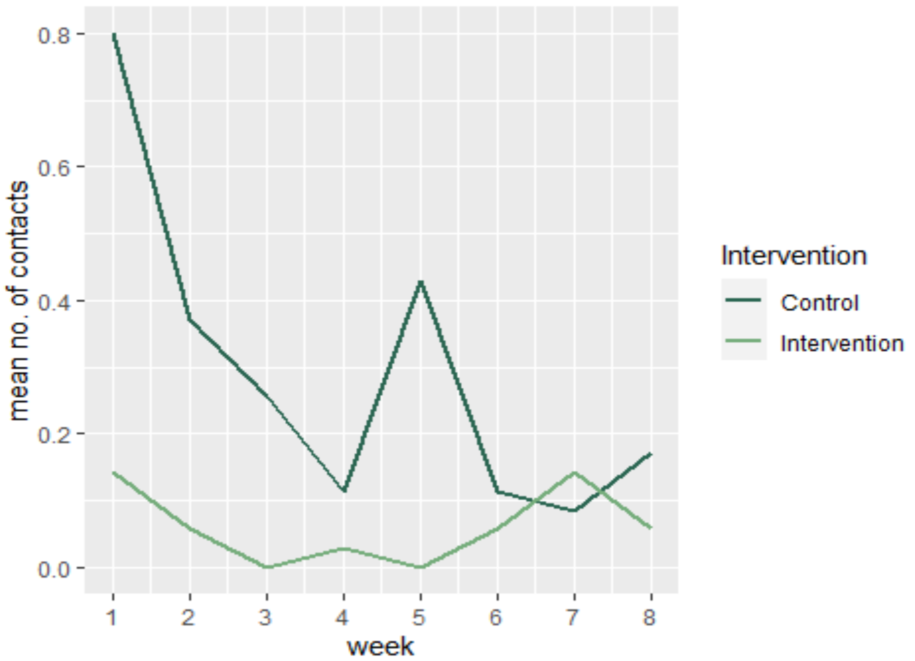

Figure 2. The mean number of phone calls per week by groups

Full report of PCCQ

Control group

Question number	Response categories, values are n (%)						
	1	2	3	4	5	N/A	Total
**Before discharge**							
pccq_1	0 (0.0)	0 (0.0)	0 (0.0)	4 (11.4)	31 (88.6)	0 (0.0)	35
pccq_2	2 (5.7)	1 (2.9)	0 (0.0)	10 (28.6)	22 (62.9)	0 (0.0)	35
pccq_3	1 (2.9)	2 (5.7)	1 (2.9)	4 (11.4)	26 (74.3)	1 (2.9)	35
pccq_4	1 (2.9)	2 (5.7)	3 (8.6)	3 (8.6)	24 (68.6)	2 (5.7)	35
pccq_5	1 (2.9)	1 (2.9)	1 (2.9)	5 (14.3)	13 (37.1)	14 (40.0)	35
pccq_6	1 (2.9)	2 (5.7)	1 (2.9)	4 (11.4)	27 (77.1)	0 (0.0)	35
pccq_7	5 (14.3)	2 (5.7)	3 (8.6)	0 (0.0)	2 (5.7)	23 (65.7)	35
pccq_8	0 (0.0)	1 (2.9)	0 (0.0)	5 (14.3)	29 (82.9)	0 (0.0)	35
pccq_9	0 (0.0)	1 (2.9)	0 (0.0)	5 (14.3)	29 (82.9)	0 (0.0)	35
pccq_10	1 (2.9)	0 (0.0)	2 (5.7)	3 (8.6)	28 (80.0)	1 (2.9)	35
pccq_11	1 (2.9)	1 (2.9)	4 (11.4)	4 (11.4)	23 (65.7)	2 (5.7)	35
pccq_12	0 (0.0)	0 (0.0)	1 (2.9)	8 (22.9)	21 (60.0)	5 (14.3)	35
pccq_13	3 (8.6)	0 (0.0)	1 (2.9)	0 (0.0)	6 (17.1)	25 (71.4)	35
pccq_14	5 (14.3)	2 (5.7)	4 (11.4)	3 (8.6)	6 (17.1)	15 (42.9)	35
pccq_15	5 (14.3)	1 (2.9)	2 (5.7)	4 (11.4)	6 (17.1)	17 (48.6)	35
pccq_16	1 (2.9)	2 (5.7)	1 (2.9)	6 (17.1)	18 (51.4)	7 (20.0)	35
pccq_17	0 (0.0)	0 (0.0)	1 (2.9)	5 (14.3)	27 (77.1)	2 (5.7)	35
pccq_18	0 (0.0)	0 (0.0)	0 (0.0)	4 (11.4)	31 (88.6)	0 (0.0)	35
pccq_19	0 (0.0)	0 (0.0)	0 (0.0)	4 (11.4)	31 (88.6)	0 (0.0)	35
pccq_20	0 (0.0)	0 (0.0)	0 (0.0)	3 (8.6)	32 (91.4)	0 (0.0)	35
pccq_21	0 (0.0)	0 (0.0)	0 (0.0)	5 (14.3)	28 (80.0)	2 (5.7)	35
pccq_22	0 (0.0)	0 (0.0)	0 (0.0)	2 (5.7)	33 (94.3)	0 (0.0)	35
pccq_23	0 (0.0)	1 (2.9)	2 (5.7)	9 (25.7)	23 (65.7)	0 (0.0)	35
pccq_24	1 (2.9)	1 (2.9)	1 (2.9)	2 (5.7)	30 (85.7)	0 (0.0)	35
pccq_25	0 (0.0)	0 (0.0)	2 (5.7)	2 (5.7)	30 (85.7)	1 (2.9)	35
pccq_26	1 (2.9)	2 (5.7)	1 (2.9)	2 (5.7)	23 (65.7)	6 (17.1)	35
pccq_27	0 (0.0)	0 (0.0)	0 (0.0)	4 (11.4)	31 (88.6)	0 (0.0)	35
**After discharge**							
pccq_28	0 (0.0)	1 (2.9)	3 (8.6)	13 (37.1)	16 (45.7)	2 (5.7)	35
pccq_29	0 (0.0)	1 (2.9)	2 (5.7)	11 (31.4)	19 (54.3)	2 (5.7)	35
pccq_30	1 (2.9)	1 (2.9)	4 (11.4)	10 (28.6)	17 (48.6)	2 (5.7)	35
pccq_31	1 (2.9)	1 (2.9)	4 (11.4)	6 (17.1)	14 (40.0)	9 (25.7)	35
pccq_32	1 (2.9)	1 (2.9)	3 (8.6)	8 (22.9)	20 (57.1)	2 (5.7)	35
pccq_33	1 (2.9)	1 (2.9)	8 (22.9)	11 (31.4)	11 (31.4)	3 (8.6)	35
pccq_34	1 (2.9)	2 (5.7)	10(28.6)	5 (14.3)	8 (22.9)	9 (25.7)	35
pccq_35	10 (28.6)	2 (5.7)	5 (14.3)	1 (2.9)	1 (2.9)	16 (45.7)	35

pccq_36	1 (2.9)	2 (5.7)	1 (2.9)	13 (37.1)	15 (42.9)	3 (8.6)	35
pccq_37	1 (2.9)	1 (2.9)	5 (14.3)	10 (28.6)	13 (37.1)	5 (14.3)	35
pccq_38	0 (0.0)	0 (0.0)	0 (0.0)	15 (42.9)	18 (51.4)	2 (5.7)	35
pccq_39	1 (2.9)	0 (0.0)	4 (11.4)	10 (28.6)	15 (42.9)	5 (14.3)	35
pccq_40	1 (2.9)	1 (2.9)	6 (17.1)	13 (37.1)	12 (34.3)	2 (5.7)	35
pccq_41	1 (2.9)	3 (8.6)	7 (20.0)	10 (28.6)	11 (31.4)	3 (8.6)	35

Intervention group

Question number	Response categories, values are n (%)						
	1	2	3	4	5	N/A	Total
**Before discharge**							
pccq_1	0 (0.0)	2 (5.7)	1 (2.9)	5 (14.3)	27 (77.1)	0 (0.0)	35
pccq_2	0 (0.0)	0 (0.0)	4 (11.4)	7 (20.0)	24 (68.6)	0 (0.0)	35
pccq_3	1 (2.9)	2 (5.7)	2 (5.7)	8 (22.9)	17 (48.6)	5 (14.3)	35
pccq_4	2 (5.7)	3 (8.6)	4 (11.4)	3 (8.6)	22 (62.9)	1 (2.9)	35
pccq_5	0 (0.0)	1 (2.9)	3 (8.6)	3 (8.6)	15 (42.9)	13 (37.1)	35
pccq_6	0 (0.0)	1 (2.9)	1 (2.9)	7 (20.0)	22 (62.9)	4 (11.4)	35
pccq_7	5 (14.3)	2 (5.7)	1 (2.9)	3 (8.6)	3 (8.6)	21 (60.0)	35
pccq_8	1 (2.9)	0 (0.0)	4 (11.4)	2 (5.7)	28 (80.0)	0 (0.0)	35
pccq_9	0 (0.0)	1 (2.9)	1 (2.9)	4 (11.4)	27 (77.1)	2 (5.7)	35
pccq_10	1 (2.9)	0 (0.0)	0 (0.0)	5 (14.3)	27 (77.1)	2 (5.7)	35
pccq_11	2 (5.7)	2 (5.7)	2 (5.7)	7 (20.0)	21 (60.0)	1 (2.9)	35
pccq_12	1 (2.9)	1 (2.9)	1 (2.9)	4 (11.4)	23 (65.7)	5 (14.3)	35
pccq_13	7 (20.0)	0 (0.0)	3 (8.6)	2 (5.7)	2 (5.7)	21 (60.0)	35
pccq_14	6 (17.1)	2 (5.7)	3 (8.6)	1 (2.9)	10 (28.6)	13 (37.1)	35
pccq_15	7 (20.0)	1 (2.9)	3 (8.6)	0 (0.0)	8 (22.9)	16 (45.7)	35
pccq_16	3 (8.6)	1 (2.9)	3 (8.6)	7 (20.0)	10 (28.6)	11 (31.4)	35
pccq_17	0 (0.0)	0 (0.0)	2 (5.7)	8 (22.9)	24 (68.6)	1 (2.9)	35
pccq_18	0 (0.0)	0 (0.0)	1 (2.9)	10 (28.6)	24 (68.6)	0 (0.0)	35
pccq_19	0 (0.0)	0 (0.0)	0 (0.0)	8 (22.9)	27 (77.1)	0 (0.0)	35
pccq_20	0 (0.0)	2 (5.7)	0 (0.0)	6 (17.1)	27 (77.1)	0 (0.0)	35
pccq_21	1 (2.9)	0 (0.0)	1 (2.9)	6 (17.1)	25 (71.4)	2 (5.7)	35
pccq_22	1 (2.9)	1 (2.9)	0 (0.0)	9 (25.7)	24 (68.6)	0 (0.0)	35
pccq_23	1 (2.9)	3 (8.6)	4 (11.4)	7 (20.0)	20 (57.1)	0 (0.0)	35
pccq_24	1 (2.9)	2 (5.7)	0 (0.0)	5 (14.3)	27 (77.1)	0 (0.0)	35
pccq_25	0 (0.0)	1 (2.9)	1 (2.9)	2 (5.7)	28 (80.0)	3 (8.6)	35
pccq_26	2 (5.7)	3 (8.6)	4 (11.4)	3 (8.6)	11 (31.4)	12 (34.3)	35
pccq_27	0 (0.0)	0 (0.0)	2 (5.7)	5 (14.3)	28 (80.0)	0 (0.0)	35
**After discharge**							
pccq_28	0 (0.0)	2 (5.7)	2 (5.7)	10 (28.6)	19 (54.3)	2 (5.7)	35
pccq_29	0 (0.0)	1 (2.9)	1 (2.9)	8 (22.9)	23 (65.7)	2 (5.7)	35

pccq_30	0 (0.0)	1 (2.9)	2 (5.7)	10 (28.6)	20 (57.1)	2 (5.7)	35
pccq_31	1 (2.9)	0 (0.0)	5 (14.3)	8 (22.9)	14 (40.0)	7 (20.0)	35
pccq_32	0 (0.0)	0 (0.0)	1 (2.9)	11 (31.4)	22 (62.9)	1 (2.9)	35
pccq_33	0 (0.0)	1 (2.9)	6 (17.1)	9 (25.7)	15 (42.9)	4 (11.4)	35
pccq_34	2 (5.7)	0 (0.0)	8 (22.9)	9 (25.7)	10 (28.6)	6 (17.1)	35
pccq_35	8 (22.9)	4 (11.4)	3 (8.6)	5 (14.3)	1 (2.9)	14 (40.0)	35
pccq_36	0 (0.0)	0 (0.0)	4 (11.4)	14 (40.0)	14 (40.0)	3 (8.6)	35
pccq_37	1 (2.9)	3 (8.6)	2 (5.7)	13 (37.1)	15 (42.9)	1 (2.9)	35
pccq_38	1 (2.9)	0 (0.0)	0 (0.0)	8 (22.9)	25 (71.4)	1 (2.9)	35
pccq_39	0 (0.0)	0 (0.0)	6 (17.1)	8 (22.9)	17 (48.6)	4 (11.4)	35
pccq_40	0 (0.0)	0 (0.0)	5 (14.3)	13 (37.1)	12 (34.3)	5 (14.3)	35
pccq_41	0 (0.0)	0 (0.0)	7 (20.0)	14 (40.0)	13 (37.1)	1 (2.9)	35

References

1. **Drennan J.** Cognitive interviewing: verbal data in the design and pretesting of questionnaires. J Adv Nurs 2003; 42(1): 57-63. doi: 10.1046/j.1365-2648.2003.02579.x.
2. **Hadjistavropoulos H, Biem H, Sharpe D, Bourgault-Fagnou M, Janzen J.** Patient perceptions of hospital discharge: reliability and validity of a Patient Continuity of Care Questionnaire. Int J Qual Health Care 2008; 20(5): 314-23. doi: 10.1093/intqhc/mzn030.
3. **Haggerty J, Reid R, Freeman G K, Starfield B H, Adair C E, McKendry R.** Continuity of care: a multidisciplinary review. Br Med J 2003; 327(7425): 1219–21. doi: 10.1136/bmj.327.7425.1219.
4. **Säfström E, Nasstrom L, Liljeroos M, Nordgren L, Årestedt K, Jaarsma T, et al.** Patient Continuity of Care Questionnaire in a cardiac sample: a confirmatory factor analysis. BMJ Open 2020; 10(7): e037129. doi: 10.1136/bmjopen-2020-037129.
5. **Wild D, Alyson G, Martin M, Eremenco S, McElroy S, Verjee-Lorenz A, et al.** Principles of good practice for the translation and cultural adaptation process for patient-reported outcomes (PRO) measures: report of the ISPOR Task Force for Translation and Cultural Adaptation. Value Health 2005 ;8(2): 94-104. doi: 10.1111/j.1524-4733.2005.04054.x.
